# An In Vitro Study on the Application of Silver-Doped Platelet-Rich Plasma in the Prevention of Post-Implant-Associated Infections

**DOI:** 10.3390/ijms25094842

**Published:** 2024-04-29

**Authors:** Chiara Ceresa, Fabio Travagin, Alice Marchetti, Francesco Tessarolo, Letizia Fracchia, Giovanni Battista Giovenzana, Michela Bosetti

**Affiliations:** 1 Department of Pharmaceutical Sciences, Università del Piemonte Orientale “A. Avogadro”, 28100 Novara, Italy; chiara.ceresa@uniupo.it (C.C.); fabio.travagin@uniupo.it (F.T.); alice.marchetti@uniupo.it (A.M.); giovannibattista.giovenzana@uniupo.it (G.B.G.); 2 Department of Industrial Engineering & BIOtech, University of Trento, 38123 Trento, Italy; francesco.tessarolo@unitn.it

**Keywords:** platelet-rich plasma, silver, scaffold, bone repair, antimicrobial activity, biofilm formation, biocompatibility, osteoblast differentiation

## Abstract

Implant therapy is a common treatment option in dentistry and orthopedics, but its application is often associated with an increased risk of microbial contamination of the implant surfaces that cause bone tissue impairment. This study aims to develop two silver-enriched platelet-rich plasma (PRP) multifunctional scaffolds active at the same time in preventing implant-associated infections and stimulating bone regeneration. Commercial silver lactate (L) and newly synthesized silver deoxycholate:β-Cyclodextrin (B), were studied in vitro. Initially, the antimicrobial activity of the two silver soluble forms and the PRP enriched with the two silver forms has been studied on microbial planktonic cells. At the same time, the biocompatibility of silver-enriched PRPs has been assessed by an MTT test on human primary osteoblasts (hOBs). Afterwards, an investigation was conducted to evaluate the activity of selected concentrations and forms of silver-enriched PRPs in inhibiting microbial biofilm formation and stimulating hOB differentiation. PRP-L (0.3 µg/mm^2^) and PRP-B (0.2 µg/mm^2^) counteract *Staphylococcus aureus*, *Staphylococcus epidermidis* and *Candida albicans* planktonic cell growth and biofilm formation, preserving hOB viability without interfering with their differentiation capability. Overall, the results obtained suggest that L- and B-enriched PRPs represent a promising preventive strategy against biofilm-related implant infections and demonstrate a new silver formulation that, together with increasing fibrin binding protecting silver in truncated cone-shaped cyclic oligosaccharides, achieved comparable inhibitory results on prokaryotic cells at a lower concentration.

## 1. Introduction

Advances in dentistry and orthopedics have allowed implant therapy to become a common clinical resource that has revolutionized modern medicine and significantly increased the quality of life of patients [1,2,3]. Although the biomaterials used are specifically designed to interact with biological systems, sometimes the implants do not integrate adequately with the bone due to septic processes in the peri-implant space that delay healing and compromise the primary stability of the implant, ending, in many cases, with implant failure [4,5,6]. Implant-associated infections pose significant challenges in treatment due to rising drug-resistant pathogens and the formation of biofilm on implant surfaces, which reduces susceptibility to antimicrobial therapy and immune responses [7,8,9,10,11]. Furthermore, bone metabolism, needed to integrate the prosthetic biomaterial, is negatively influenced by the immune system, which plays a pivotal role during infection. Immune cells and their inflammatory cytokines, such as TNF-α, IL-1β, and IL-17, adversely affect bone cells, such as osteoblasts and osteoclasts, impairing matrix deposition, bone metabolism, and density. This process upregulates RANK-L and M-CSF expression, which increases the number of osteoclasts and enhances their phagocytic activity, leading to bone resorption [12,13,14]. Staphylococci are the most common opportunistic pathogens responsible for these infections, with *Staphylococcus aureus* and *Staphylococcus epidermidis* as the leading causes, but other bacteria and fungi, especially *Candida albicans*, are also involved [9,15,16,17,18].

Despite the high frequency of bone infections, a precise standardized approach to cope with this eventuality is lacking. In most cases, infection management involves soft tissue coverage and both local and systemic administration of antibiotics; usually, first-generation cephalosporins are recommended [19], while penicillin and clindamycin are used as prophylaxis [12]. The use of systemic antibiotics brings some disadvantages, though. Bacteria resistance is the first concern, especially because relapses are very common and more complicated to handle. The micro-environment of infected bones is usually impervious to systemic antibiotics due to impaired vasculature, which is the result of a high inflammation process. Consequently, subtherapeutic levels of antibiotics systemically administered could lead to bacterial resistance development. To avoid this, the antibiotic dose required for effective penetration of bone tissue and desired therapeutic effect is typically increased, which frequently leads to bone or other tissue toxicity [20]. Moreover, Hathaway-Schrader et al. [21] found that systemic antibiotics alter gut microbiota, impacting bone metabolism. Antibiotics reduce osteoblast ALP activity while increasing osteoclast number, size, and activity, resulting in significant metabolic and structural changes in trabecular bone [13]. In the above-mentioned context, the local administration of antimicrobial compounds could represent a valid alternative to address these challenges and avoid bone prosthetic failure.

One approach involves enhancing a bone bioactive material with an antimicrobial agent, either encasing the implant or blending it with bone substitutes. This strategy could be more effective than solely administering local injections of antimicrobials, as it offers a dual benefit by simultaneously promoting bone regeneration and combating infection. Indeed, many of the biomaterials already used in orthopedic applications such as ceramics, collagen, poly-lactic acid, polymethyl methacrylate, bioglasses, titanium, resins, and cements are industrially imbued or coated with antimicrobial compounds. These can be antibiotics (i.e., gentamicin, tobramycin, vancomycin, and clindamycin) [22] or non-antibiotic compounds, such as bio-surfactants [23], nanoparticles (silver or titanium) [24], or metallic compounds. In particular, silver (as silver salts, nanoparticles, or metallic silver), whose antimicrobial properties have been known for decades [25,26], is widely used as an antimicrobial-enriching compound for biomaterials [27]. Starting from the XVII and XVIII centuries, silver has been widely used for infection prevention and wound treatment [28]. Lately, the growing development of bacterial resistance to most antibiotics has led to a reexamining of its potential because of its broad spectrum of action and the rare resistance cases associated [29]. The exact antimicrobial mechanism remains uncertain but likely involves generating free radicals and reactive oxygen species, inactivating vital enzymes via thiol group interactions, and bonding with bacterial surface membranes, potentially leading to cell lysis [30].

With regard to scaffolds with bone regenerative potentials, platelet-rich plasma (PRP) is an autologous blood derivative consisting of highly concentrated platelets rich in growth factors that can be embedded in its 3D fibrin matrix after clotting and utilized in different regenerative medicine treatments [31]. Acting like a depot of growth factors, it is exploited in oral surgery, dentistry [32,33,34,35,36], and orthopedic surgery [32,37], where it accelerates bone regeneration [37,38,39], enhances bone formation, increases bone density [34], and augments cortical bone thickness [32]. In vitro studies proved that PRP enhances osteoblast proliferation [40,41,42,43], adhesion to a membrane surface [44], migration [45], and differentiation [44,46,47], with higher mRNA levels of ALP, Runx2, and COL1α2 [40] detected after PRP treatment. Moreover, PRP enhances osseointegration around various implant materials (such as ceramics, glasses, titanium implants, or bone grafts), accelerating early bone formation and improving implant stability, thus enhancing overall treatment success [48,49,50,51]. This evidence has opened up new opportunities, from augmenting bone volume for implant placement to facilitating post-implant surgery healing, promising improved outcomes in dental and orthopedic bone regeneration procedures.

Starting from all these considerations, this study aimed to create two silver-enriched PRP multifunctional scaffolds active both in preventing implant-associated infections and stimulating bone regeneration. To satisfy this purpose, we initially designed and synthesized a novel silver-containing compound with a high affinity for fibrin clots (silver deoxycholate:β-Cyclodextrin) and, then, investigated (1) its in vitro antimicrobial activity against staphylococcal/yeast planktonic cells by comparing it to a commercial alternative silver compound (silver lactate) and studying them in their soluble form and PRP silver-enriched clusters; (2) the biocompatibility of the two forms of silver-enriched PRP clusters using human primary osteoblasts (hOBs); (3) their activity in inhibiting biofilm formation and (4) in promoting hOB differentiation.

## 2. Results

### 2.1. Silver-Containing Compounds

Silver lactate (L) is commercially available and used in infection treatment, whereas the newly synthesized silver deoxycholate:β-Cyclodextrin (B), was designed to improve the interaction of the silver compound with the fibrin clot.

**Figure 1:**
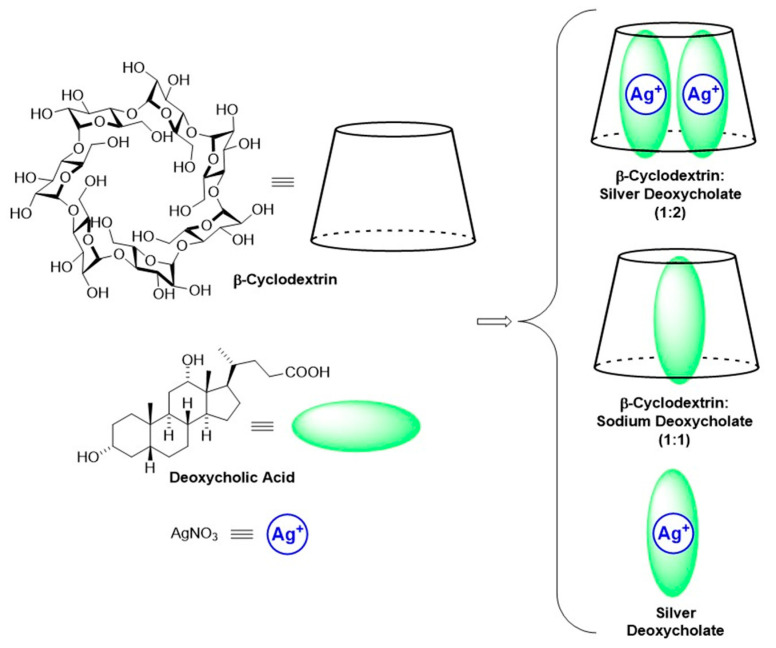
Cyclodextrin inclusion complexes.

Cyclodextrins are truncated cone-shaped cyclic oligosaccharides whose ability to host molecules in their cavity has been widely exploited for the vehiculation of bioactive cargoes [52]. The outer surface of cyclodextrins is decorated with multiple hydroxyl groups, enabling them to interact with biomacromolecules by hydrogen bonding. In our intention, cyclodextrin should act as the molecular container of silver salt and show a strong interaction with the fibrin clot. The silver salt to be included must be lipophilic and suitably sized to fit in the hydrophobic cavity of cyclodextrin. Bile acids are particularly suited for this purpose as they form stable inclusion complexes with cyclodextrin [53,54]. Among them, we selected deoxycholic acid (Figure 1) to form a lipophilic silver salt (silver deoxycholate) and then formed an inclusion complex with β-Cyclodextrin, showing a 2:1 stoichiometry (β-Cyclodextrin/sodium deoxycholate, 1:2). To our knowledge, this is the first example of a supramolecular inclusion compound for the controlled delivery of silver ions, where the slow dissociation of the supramolecular assembly triggers the release of the active metal ion. A silver-free inclusion complex (sodium deoxycholate:β-Cyclodextrin 1:1) has been prepared as a reference compound, too.

### 2.2. Morphological Characterization of PRP Clusters

At low magnification (100×), PRP clusters subjected to dehydration and drying resulted in a bristling disk-shaped structure in agreement with what was reported in [55]. Porous structures were visible in between more compact and amorphous areas in all Ag-enriched samples and in samples without any enrichment. Microstructural characterization at higher magnifications was then focused on areas showing interconnected structures. A representative selection of high-magnification SEM images of both non-enriched and Ag-enriched PRP clusters is presented in Supplementary Materials (Figure S1). Overall, the samples’ microstructure was mostly characterized by platelet aggregates, which almost completely hid the underlying fibrin structure. Platelet shape and size were found to be comparable among samples. Thin fibrin fibers and fibrin networks [56] were occasionally identified, preferably in PRP enriched with silver deoxycholate:β-Cyclodextrin.

### 2.3. Antimicrobial Activity of Silver Solutions on Planktonic Cells

The two silver compounds (L and B) were first tested in a free-soluble form. The antimicrobial activity of solutions against bacterial or yeast planktonic cells was evaluated and compared by the 3-[4,5-dimethylthiazol-2-yl]-2,5-diphenyltetrazolium bromide (MTT)-based colorimetric assay. The concentrations tested, expressed as µg of silver inside PRP clusters (mm^2^), were selected with regard to our previous study on alginate and collagen clusters enriched with L [57] and ranged from 0.06 µg/mm^2^ to 0.3 µg/mm^2^.

**Figure 2:**
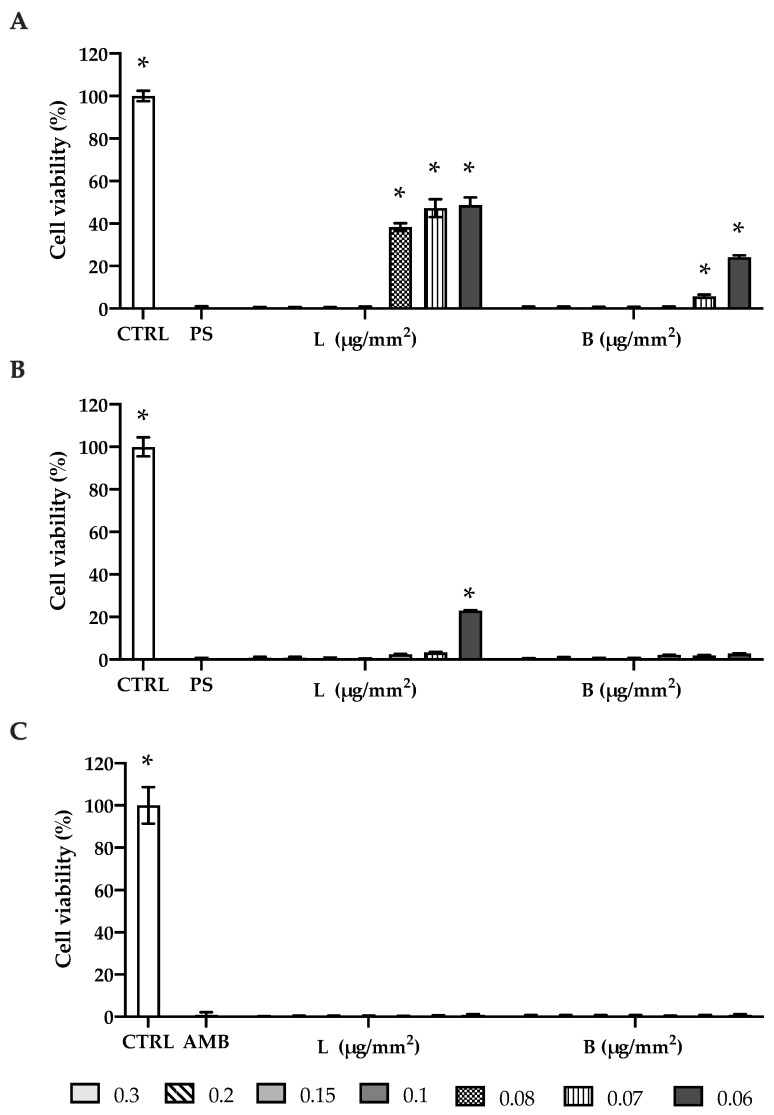
Effect of silver solutions on the viability of microbial planktonic cells. The metabolic activity of *S. aureus* (**A**), *S. epidermidis* (**B**), and *C. albicans* (**C**) planktonic cells was evaluated after 24 h of exposure to silver solutions through an MTT assay. Data are expressed as a percentage of cell viability, considering CTRL (positive control) as 100% vitality (white bar). Pen Strep (PS) or amphotericin B (AMB) were used as negative controls. CTRL: not-treated microbial cells; PS: treatment with PS; AMB: treatment with AMB; L: treatment with silver lactate at different concentrations; B: treatment with silver deoxycholate:β-Cyclodextrin at different concentrations. Error bars represent standard deviation, * *p* < 0.0001 significantly different from PS/AMB.

Results are shown in Figure 2. At higher concentrations (0.1–0.3 µg/mm^2^), L showed a remarkable concentration-independent antibacterial activity on *S. aureus* (Figure 2A), with a viability reduction in bacteria growth of 99%, similar to negative controls, Pen Strep (PS *p* > 0.05); at lower concentrations (0.06–0.08 µg/mm^2^), its efficacy, although significant (*p* < 0.001), decreased with viability reductions of 50–60% when compared to CTRL. At the same concentrations, B evidenced a more pronounced bactericidal effect than L (*p* < 0.001) with viability reductions of 75–95% (0.06–0.07 µg/mm^2^). In addition, a lower concentration of B was required to totally inhibit cell viability (0.08 µg/mm^2^). On *S. epidermidis* (Figure 2B), both silver compounds at all the concentrations, with the exception of L at 0.06 µg/mm^2^, were able to completely kill staphylococcal cells, as observed for PS (*p* > 0.05). Similarly, L and B demonstrated a strong concentration-independent fungicidal activity on *C. albicans*, equal to that observed for amphotericin B (AMB) (Figure 2C).

As a component of B, the antimicrobial effect of sodium deoxycholate:β-Cyclodextrin 1:1, against *S. aureus* and *C. albicans* was checked. The data obtained (Supplementary Figure S2) demonstrated that the molecules at the concentrations tested (0.06–0.3 µg/mm^2^) did not affect the growth of the two strains, suggesting that the antimicrobial activity of B was due only to silver ions.

### 2.4. Effect of PRP-Ag Clusters on Microbial Planktonic Cells

**Figure 3:**
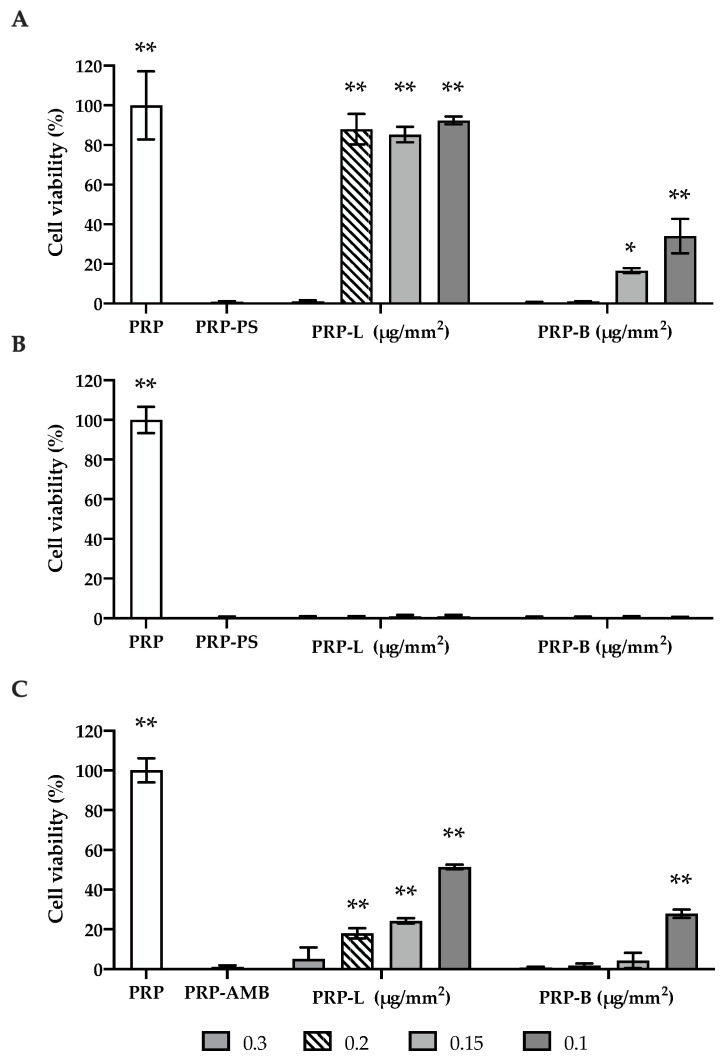
PRP-Ag clusters’ effect on the viability of microbial planktonic cells. The metabolic activity of *S. aureus* (**A**), *S. epidermidis* (**B**), and *C. albicans* (**C**) planktonic cells was evaluated after 24 h of exposure to PRP-Ag through an MTT assay. Data are expressed as a percentage of cell viability, considering PRP (positive control-white bar) as 100% vitality. PRP-PS or PRP-AMB were used as negative controls. PRP: treatment with PRP; PRP-PS: treatment with PRP enriched with PS; PRP-AMB: treatment with PRP enriched with AMB; PRP-L: treatment with PRP enriched with silver lactate at four different concentrations; PRP-B: treatment with PRP enriched with silver deoxycholate:β-Cyclodextrin at four different concentrations. Error bars represent standard deviation; * *p* < 0.01 and ** *p* < 0.0001 are significantly different from PRP-PS/PRP-AMB.

The antimicrobial activity of PRP-Ag clusters on bacterial and fungal planktonic cells was measured after 24 h through the MTT-based cell metabolic assay. The concentrations tested (from 0.1 to 0.3 µg/mm^2^) were selected with regard to the results described above as doses of Ag able to completely kill the microbial strains. Results are shown in Figure 3. On *S. aureus* (Figure 3A) and *C. albicans* (Figure 3C), PRP-L at 0.3 µg/mm^2^ was able to highly reduce microbial cell viability (99%), similar to the negative control PS or AMB (*p* > 0.05). Conversely, PRP-L at lower concentrations had no antibacterial activity as confirmed by Tukey’s post hoc test (no significant differences when compared to PRP, *p* > 0.05) and showed a lower, albeit significant and concentration-dependent, antifungal activity, with viability reductions ranging from 18% to 51%. As illustrated in its free-soluble form, PRP-B exhibited heightened efficacy in inhibiting the growth of *S. aureus* and *C. albicans*. This was evident as lower concentrations (0.2 µg/mm^2^, 0.15 µg/mm^2^) were sufficient to achieve a comparable effect observed with PS and AMB treatments (*p* > 0.05). Similarly, all concentrations of the PRP-L and PRP-B tested demonstrated a marked concentration-independent antibacterial activity on *S. epidermidis* (Figure 3B), equal to that observed for PS.

Notably, a preliminary study about the antimicrobial activity of PRP clusters with no silver enrichment was also conducted. The data obtained (Supplementary Figure S3) did not reveal any significant inhibitory activity on microbial growth.

### 2.5. Effect of PRP-Ag Clusters on hOB Viability

**Figure 4:**
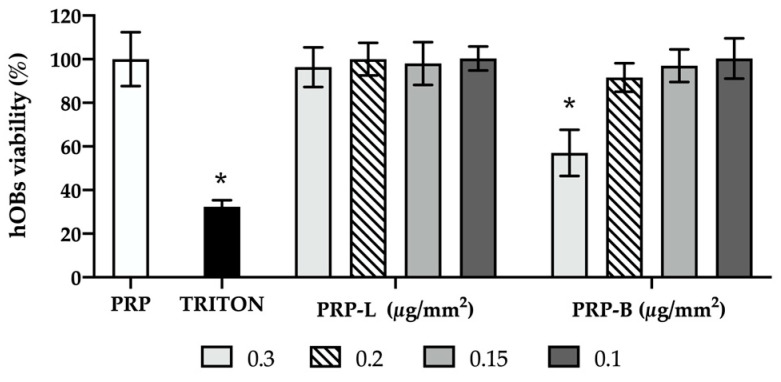
PRP-Ag clusters’ effect on hOB viability. The metabolic activity of hOB cells was evaluated after 24 h of exposure to PRP-Ag through an MTT assay. Data are expressed as a percentage of cell viability, considering PRP (positive control-white bar) as 100% vitality. Triton-X was used as a negative control (black bar). PRP: hOBs co-cultured with PRP; TRITON: hOBs treated with Triton-X; PRP-L: hOBs co-cultured with PRP enriched with silver lactate at four different concentrations; PRP-B: hOBs co-cultured with PRP enriched with silver deoxycholate:β-Cyclodextrin at four different concentrations. Error bars represent standard deviation; * *p* < 0.0001 is significantly different from PRP.

hOB viability was assessed after 24 h of exposure to PRP-Ag clusters through the MTT test. Results are shown in Figure 4. Triton-X was used as a negative control of cell viability. All PRP-L clusters (0.3, 0.2, 0.15, 0.1 µg/mm^2^) maintained a high hOB viability over 90%, with no significant differences among the concentrations tested (*p* > 0.05). Similarly, PRP-B clusters (0.2, 0.15, 0.1 µg/mm^2^) did not affect hOB viability (values over 90% of cell vitality, *p* > 0.05). In contrast, clusters of PRP-B at 0.3 µg/mm^2^ exhibited a reduction in hOB viability, amounting to approximately 57% (*p* < 0.0001).

### 2.6. Effect of PRP-Ag Clusters on Biofilm Formation

Taking into account the results obtained about the antimicrobial efficacy of PRP-Ag and their biocompatibility, PRP-L (0.3 µg/mm^2^) and PRP-B (0.2 µg/mm^2^) were chosen for subsequent assays, as these clusters resulted to be enriched with the lowest concentration of L or B able to inhibit the microbial growth (≥90%) for all tested strains, preserving hOB vitality.

**Figure 5:**
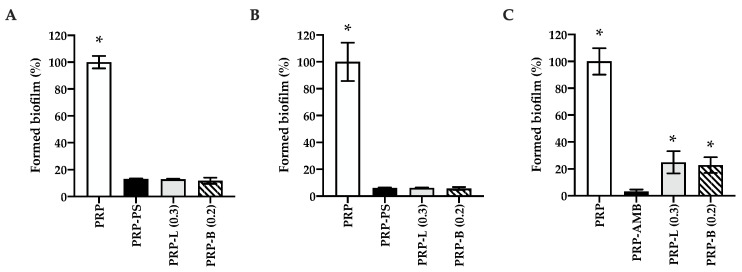
PRP-Ag effect on biofilm formation. Metabolic activity of the cells forming the microbial biofilms evaluated after 24 h of exposure to PRP-Ag by the MTT test: (**A**) *S. aureus*, (**B**) *S. epidermidis*, (**C**) *C. albicans*. Data are expressed as a percentage of formed biofilm normalized to PRP, considered as a positive control (white bar), corresponding to 100%. PRP-PS or PRP-AMB were used as negative controls. PRP: treatment with PRP; PRP-PS: treatment with PRP enriched with PS; PRP-AMB: treatment with PRP enriched with AMB; PRP-L (0.3): treatment with PRP enriched with silver lactate (0.3 µg/mm^2^); PRP-B (0.2): treatment with PRP enriched with silver deoxycholate:β-Cyclodextrin (0.2 µg/mm^2^). Error bars represent standard deviation; * *p* < 0.0001 is significantly different from PRP-PS/PRP-AMB.

The inhibition of bacterial and fungal biofilm formation by selected PRP-Ag was assessed after 24 h of exposure through the MTT test. Results are shown in Figure 5. PRP-PS and PRP-AMB were used as negative controls of staphylococcal and fungal biofilm growth, respectively. In general, PRP-Ag showed an interesting antibiofilm activity against all the microbial strains. In particular, PRP-L (0.3 µg/mm^2^) and PRP-B (0.2 µg/mm^2^) markedly limited staphylococcal biofilm formation (*p* < 0.0001) when compared to the positive control (PRP) with reductions of about 87% and 94% for *S. aureus* (Figure 5A) and *S. epidermidis* (Figure 5B), respectively. Concerning *C. albicans*, a significant inhibitory activity of PRP-Ag on biofilm formation was also observed (*p* < 0.0001) when compared to PRP, with a reduction of about 76% (Figure 5C). In addition, for all the microbial strains, no differences were observed between the inhibition values obtained for PRP-L and PRP-B (*p* > 0.05).

### 2.7. PRP-Ag Clusters’ Effect on hOB Differentiation

**Figure 6:**
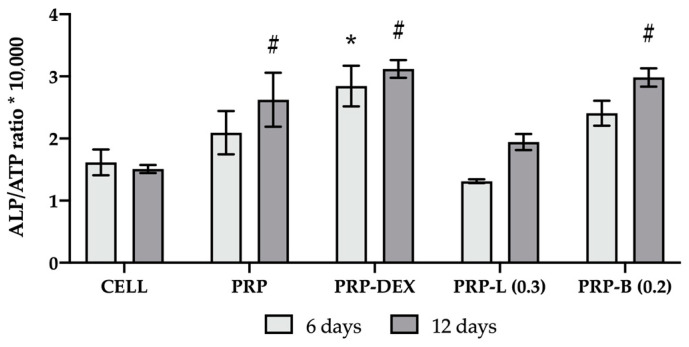
PRP-Ag differentiation activity on hOBs. Data are expressed as the ALP/ATP ratio*10,000 after 6 and 12 days of co-culture. For each condition, ALP values were normalized to the number of cells by ATP quantification. CELL: not-treated hOBs; PRP: treatment with PRP; PRP-DEX: treatment with PRP enriched with dexamethasone; PRP-L (0.3): treatment with PRP enriched with silver lactate (0.3 µg/mm^2^); PRP-B (0.2): treatment with PRP enriched with silver deoxycholate β-Cyclodextrin (0.2 µg/mm^2^). Error bars represent standard deviation; * *p* < 0.01 is significantly different from CELL after 6 days, and ^#^ *p* < 0.001 is significantly different from CELL after 12 days.

The activity of PRP-Ag clusters on hOB differentiation was investigated to clarify whether the presence of silver could interfere with the positive effect of PRP in bone regeneration. Alkaline phosphatase (ALP) activity was quantified for hOB cells co-cultured with PRP-L 0.3 µg/mm^2^ and PRP-B 0.2 µg/mm^2^ after 6 and 12 days. Data shown in Figure 6 are normalized to adenosine triphosphate (ATP) level and expressed as an ALP/ATP ratio*10.000.

Not-treated hOBs, PRP, and PRP enriched with dexamethasone were used, respectively, as the control of the basal condition (CELL), PRP effect (PRP), and differentiative activity induced by dexamethasone (PRP-DEX). Our PRP data confirmed other in vitro studies in the literature [47,58], enhancing hOB differentiation with a significant difference at day 12 (*p* < 0.05) compared to the cells with no treatment (CELL). DEX addition to PRP showed no further increase at day 12 compared to PRP and evidenced only a faster differentiation with an increased value of the ALP/ATP ratio at 6 days of culture when compared to CELL.

PRP-B compounds did not negatively affect hOB differentiation and did not show any differences with PRP and PRP-DEX values at both time points (*p* > 0.05), maintaining their statistically higher activity when compared to CELL after 12 days (*p* < 0.001). Instead, PRP-L (0.3 µg/mm^2^)-treated hOB showed ALP values normalized to ATP similar to the basal control (CELL) at both time points (*p* > 0.05) and statistically different from PRP and PRP-B at 6 days, whereas at 12 days, the gap between PRP and PRP-L decreases with no significant differences. Overall, although both compounds proved not to negatively affect osteoblast proliferation (ATP results), the difference between B and L in ALP/ATP ratio values is still significant.

## 3. Discussion

While biomaterials are intentionally designed for interaction and integration with biological systems, they must be studied, and solutions must be found when implants fail to adequately integrate with bone tissue. Septic and aseptic processes in the peri-implant space can impede healing, threatening implant stability and, in severe cases, necessitating removal and substitution. Implant-associated infections pose significant challenges in treatment due to the growing prevalence of drug-resistant pathogens. Moreover, these infections can develop biofilms on implant surfaces, rendering them more resistant to antimicrobial therapy and less susceptible to host immune responses, leading to increased patient morbidity and contributing to elevated healthcare system costs [7,9,10,11]. Despite the frequent occurrence of bone infections, there is a lack of precise and standardized approaches for their management. Typically, soft tissue coverage and the administration of local and systemic antibiotics are employed. One approach involves enhancing a bone bioactive material with antimicrobial substances, a strategy that combines benefits for bone regeneration and infection control.

In this study, we suggest a new strategy for bone regeneration and prosthesis–bone integration, which could enhance bone healing and prevent various microbial infections. We propose autologous blood derivate (PRP) as a scaffold naturally rich in growth factor, enriched with a new silver compound easily added during clot formation as an antimicrobial and antibiofilm agent to be clinically added and mixed with bone fragment substitutes or at the bone/prosthesis interface.

The antimicrobial activity of PRP has been previously investigated by some authors to understand whether this biological scaffold has its own ability to negatively affect microbial growth [59,60]. Although some in vitro studies demonstrated an antibacterial effect of PRP on different bacterial species in short-time incubation experiments, when co-incubated for 24 h, we did not observe any significant differences in metabolic activity between non-treated microbial cells and bacteria/fungi incubated together with PRP (Supplementary Figure S3), in accordance with the observations reported by other researchers [59,60,61,62]. Moreover, the pronounced variability inherent in the manufacturing protocol of platelet-rich plasma (i.e., the activation state of platelets, platelet concentration, the presence of leukocytes) along with patient-specific variations (i.e., individual platelet counts, growth factors released, the influence of drugs affecting platelets) may account for the observed conflicting results.

Our study demonstrated that boosting PRP with silver could meet clinical needs. Silver lactate is a polar molecule that can be entrapped into the fibrin clot because of its polarity. Its small size may contribute to its loss during clot harvesting due to a phenomenon of clot shrinkage that could explain our results that showed that in the solution (Figure 2A) the transition from nearly complete inhibition (99%) to partial inhibition occurs between concentrations of 0.1 µg/mm^2^ and 0.08 µg/mm^2^. Differently, for PRP-L clusters (Figure 3A), the change in trend takes place between concentrations of 0.3 µg/mm^2^ and 0.2 µg/mm^2^; thus, the initial PRP-L concentration yielding 100% inhibition is 0.3 µg/mm^2^. A direct comparison between Ag solutions and PRP-Ag clusters in terms of the antimicrobial effect on planktonic cells could be helpful in understanding how much silver was actually delivered into the scaffolds. Indeed, a correct quantification of PRP silver content is fundamental to better understanding the results obtained in this study. Unfortunately, precise silver quantification requires specific analysis such as inductively coupled plasma mass spectroscopy [63], furnace atomic absorption spectrophotometer [64], or neutron activation analysis [65] that were not available at the moment of the investigation. Nevertheless, we can speculate regarding the Ag content within PRP clusters by considering the outcomes observed with *S. aureus*. Being the least susceptible, this strain exhibited substantial variations in microbial viability in response to treatments with varying Ag concentrations. In consideration of this, we can provide a rough estimation of the silver content within the clusters. The silver quantity delivered with PRP-L at 0.3 µg/mm^2^ is approximately equivalent to the silver administered with a 0.1 µg/mm^2^ solution, constituting approximately 33% of the amount used to dope the cluster.

Similar considerations can be made regarding the B compound. PRP-B 0.1 µg/mm^2^ corresponds in terms of antimicrobial activity to 0.06 µg/mm^2^ silver solution; hence, the amount of silver actually delivered by the cluster seems to be 60% of the total. The β-Cyclodextrin of the silver deoxycholate:β-Cyclodextrin compound was selected because it is largely used as a vehicle for the delivery of many substances, in addition to its structure being very bulky and the carried molecules being better entrapped into the fibrin net of the clot. In addition, the presence of many -OH groups could bind with the polar interaction of the fibrin proteins. The difference in the amount of silver delivered could be explained by the different chemistry of the two molecules. Another factor lies in solvents used to solubilize the two compounds: DMSO for B and water for L. DMSO influences the morphological and mechanical properties of the PRP-B cluster, which appeared discoidal, flattened, thicker, and more gelatinous compared to PRP-L, leading to less plasma residues during clot formation and, hence, a lower amount of silver loss during the clotting procedure.

Despite the wide use of silver and other antimicrobial agents with a broad spectrum of activity against different pathogens, silver ions can exert toxic effects on human cell lines, impairing their viability [66,67,68]. Hence, PRP-Ag biocompatibility was investigated on human primary osteoblasts, as a major cell type that could be affected by silver toxicity. To achieve this, hOB viability was assessed after 24 h of exposure to PRP-Ag clusters through the MTT test. Consistent with the guidelines set by the International Organization for Standardization for assessing medical devices through in vitro cytotoxicity, a threshold of 70% cell viability is considered acceptable. All PRP-L clusters maintained a high hOB viability of over 90%, with no significant differences among the concentrations tested (*p* > 0.05). Conversely, PRP-B clusters at 0.3 µg/mm^2^ had a toxic effect on hOBs (*p* < 0.0001), but at lower concentrations (0.2, 0.15, 0.1 µg/mm^2^), they did not affect their viability (*p* > 0.05).

A few years ago, a study on silver lactate alginate hydrogel for peri-implantitis illustrated the effect of this compound on gingival mesenchymal stem cells and bone marrow mesenchymal stem cells [65]. Cell viability was quantified after 1 and 7 days of contact, and its lowest concentration was comparable to that of PRP-L 0.3 µg/mm^2^, showing similar results. Moreover, Xie et al. [69] conducted an extensive investigation into the impact of silver nanoparticles on osteoblast-like cells in terms of uptake, retention, and osteogenic activity. They demonstrated that a low dosage of silver nanoparticles (0.5 µg/mL up to 20 µg/mL) did not affect MG-63 cell viability over a 72 h exposure. However, the silver form under investigation in this study, like many others, primarily consists of nanoparticles, making a direct comparison of concentration effects challenging. Therefore, the last aspect considered was the ability of PRP-L 0.3 µg/mm^2^ and PRP-B 0.2 µg/mm^2^ to promote hOB differentiation. Because PRP is well known in the literature for its positive effect in inducing osteoblast differentiation, we investigated whether the presence of silver in the clusters could negatively affect this activity for bone regeneration. To achieve this, alkaline phosphatase (ALP) activity was quantified after 6 and 12 days of co-culture and normalized to adenosine–triphosphate (ATP) used as the proliferative index. In accordance with other in vitro studies [47,58,70], a significant increase in the ALP/ATP ratio was observed for PRP clusters when compared to not-treated cells (CELL) at day 12 (*p* < 0.01), demonstrating the positive effect of PRP on hOB differentiation. As a further confirmation, PRP-DEX clusters, considered as a positive control of differentiation, resulted in a trend similar to PRP, showing not significantly different (*p* > 0.05) values of the ALP/ATP ratio at both time points. Interestingly, PRP-B clusters (0.2 µg/mm^2^) did not affect hOB differentiation, displaying no significant difference when compared to PRP and PRP-DEX (*p* > 0.05). Conversely, PRP-L (0.3 µg/mm^2^) values were similar to the basal control (CELL) at both time points (*p* > 0.1).

In a further step, we decided to investigate the effects of PRP-L and PRP-B on staphylococcal and fungal biofilm formation, a crucial event that can exacerbate microbial infections. In their sessile form, microorganisms express a distinct phenotypic trait and become more tolerant to antimicrobial agents and host defense systems [62,71,72,73,74]. For this purpose, non-cytotoxic concentrations that provided the highest inhibition on planktonic cells were selected. Starting from this assumption, we proceeded to investigate the effects of PRP-L 0.3 µg/mm^2^ and PRP-B 0.2 µg/mm^2^ on biofilm formation. In general, both the Ag-enriched PRP tested equally inhibited the formation of microbial biofilms (*p* > 0.05); in particular, Ag-enriched PRP exhibited higher efficacy against *S. aureus* and *S. epidermidis*, resulting in a biofilm reduction comparable to PS-enriched PRP (*p* > 0.05) when compared to its efficacy against *C. albicans*. For *C. albicans*, PRP-L and PRP-B demonstrated significant inhibitory activity (*p* < 0.0001), although lower than AMB. In this context, we hypothesize that the observed outcome may stem from the ability of silver ions to concurrently impact both cell viability and the quorum sensing signaling crucial for biofilm development, as indicated by previous research [73,75,76,77,78,79,80]. These findings underscore the potential of PRP enriched with silver as an effective antibiofilm agent, warranting further exploration in clinical settings.

## 4. Materials and Methods

### 4.1. Solvents and Reagents

For the synthesis of compounds, commercially available solvents and reagents purchased from Merck (Darmstadt, Germany) or TCI Europe (Zwijndrecht, Belgium), were used without further purification. All aqueous solutions were prepared from ultrapure laboratory-grade water (18 MΩ·cm) obtained from the Millipore/MilliQ, (Darmstadt, Germany) purification system. Silver lactate, penicillin/streptomycin (PS, 100 U/mL and 100 μg/mL), and amphotericin B (250 μg/mL) were purchased from Sigma-Aldrich (Milan, Italy).

### 4.2. Spectra

^1^H and ^13^C NMR spectra were recorded at 400 MHz on a Bruker Avance Neo 400 spectrometer using Topspin 4.0.7 as acquisition software (Billerica, MA, USA). Chemical shifts are reported in ppm, with the protic impurities of the deuterated solvent as the internal reference.

### 4.3. Synthesis of Silver-Containing Compounds

The supramolecular derivative (B) has been designed for the present study to improve the interaction with PRP and the delivery of the metal ion. Silver deoxycholate and silver-free supramolecular adduct sodium deoxycholateβ-Cyclodextrin, were prepared as reference materials for the comparison with the selected silver-containing compounds (Figure 1).

#### 4.3.1. Silver Deoxycholate

A solution of silver nitrate (0.221 g, 1.30 mmol) in water (4.3 mL) was added to a solution of deoxycholic acid (0.340 g, 0.866 mmol) and sodium bicarbonate (0.109 g, 1.30 mmol) in 6.7 mL of water at room temperature (RT) with stirring. Silver deoxycholate was precipitated and isolated by vacuum filtration, washed with water, ethanol, and diethyl ether, dried over P_2_O_5_ in a desiccator overnight, and stored in an amber glass vessel in the dark.

^1^H NMR (400 MHz, DMSO-d_6_, 298 K) *δ* 4.45 (d, *J* = 4.3 Hz, 1H), 4.17 (d, *J* = 4.0 Hz, 1H), 3.79 (s, 1H), 3.35 (s 1 H), 2.19–1.98 (m, 2H), 1.83–0.95 (m, 24H), 0.91 (d, *J* = 6.3 Hz, 3H), 0.84 (s, 3H), and 0.59 (s, 3H) ppm.

#### 4.3.2. Silver Deoxycholate:β-Cyclodextrin 2:1

Deoxycholic acid (0.3926 g, 1 mmol) was dissolved in 5 mL of water, and sodium bicarbonate (0.0840 g, 1 mmol) was added. The mixture was refluxed for 5 min until a solution was obtained. The reaction was cooled to RT, β-Cyclodextrin (1.1350 g, 1 mmol) was added, and the mixture was stirred for 1 h until a clear solution was obtained. Then, the reaction flask was protected from the light and a solution of silver nitrate (0.1699 g, 1 mmol) in 1 mL of water was added dropwise for 5 min with stirring. The supramolecular complex was precipitated and isolated by centrifugation, washed three times with water and once with acetone, and dried under a vacuum to obtain a brown solid (0.321 mg, 28%).

^1^H NMR (400 MHz, DMSO-d_6_, 298 K) δ 5.76 (d, *J* = 6.4 Hz, 7H), 5.72 (s, 7H), 4.82 (d, *J* = 3.5 Hz, 7H), 4.47–4.44 (m, 9H), 4.18 (d, *J* = 4.0 Hz, 2H), 3.79 (s, 2H), 3.69–3.54 (m, 30H), 3.30–3.27 (m, 14H), 2.15–2.07 (m, 2H), 2.01–1.94 (m, 2H), 1.83–0.95 (m, 48H), 0.90 (d, *J* = 6.3 Hz, 6H), 0.84 (s, 6H), and 0.59 (s, 6H) ppm.

^13^C NMR (100 MHz, DMSO-d_6_, 298 K) δ 177.3 (C), 102.0 (CH), 81.6 (CH), 73.1 (CH), 72.5 (CH), 72.1 (CH), 71.1 (CH), 70.0 (CH), 60.0 (CH_2_), 47.5 (CH), 46.4 (CH), 46.0 (C), 41.7 (CH), 36.3 (CH_2_), 35.7 (CH), 35.3 (CH), 35.2 (CH_2_), 33.9 (C), 33.2 (CH_2_), 33.0 (CH), 32.4 (CH_2_), 30.3 (CH_2_), 28.7 (CH_2_), 27.3 (CH_2_), 27.0 (CH_2_), 26.2 (CH_2_), 23.6 (CH_2_), 23.1 (CH_3_), 17.1 (CH_3_), and 12.5 (CH_3_) ppm.

#### 4.3.3. Sodium Deoxycholate:β-Cyclodextrin 1:1

Deoxycholic acid (0.346 g, 0.881 mmol) was suspended in water (10 mL), and sodium bicarbonate (0.0740 g, 0.881 mmol) was added with stirring. The mixture was refluxed for 5 min to obtain a clear solution and then cooled to RT. β-Cyclodextrin (1.00 g, 0.881 mmol) was added, and the mixture was stirred for five min until a clear solution was obtained. The mixture was left at RT for 5 h and then dropped in 150 mL of acetone with vigorous stirring. After 15 min, the solid precipitate was vacuum filtered, washed with acetone, and dried in a vacuum to give a white solid (1.12 g, 82%).

^1^H NMR (400 MHz, D2O, 298 K) δ 5.07 (d, *J* = 2.6 Hz, 7H), 4.13 (s, 1H), 3.95 (t, *J* = 9.9 Hz, 14H), 3.87–3.81 (m, 15H), 3.67 (dd, *J* = 10.0/3.5 Hz, 7H), 3.57 (t, *J* = 9.4 Hz, 7H), 2.23–1.20 (m, 26H), 1.07 (d, *J* = 6.1 Hz, 3H), 1.01 (s, 3H), and 0.83 (s, 3H) ppm.

^13^C NMR (101 MHz, D2O, 298 K) δ 181.2 (C), 102.3 (CH), 81.7 (CH), 73.8 (CH), 73.3 (CH), 72.1 (2 CH), 71.7 (CH), 60.2 (CH2), 48.1 (CH), 47.8 (CH), 46.3 (C), 42.2 (CH), 36.9 (CH), 36.0 (CH_2_), 35.8 (CH_2_), 35.7 (CH), 35.0 (CH_2_), 33.9 (C), 33.8 (CH), 33.5 (CH_2_), 33.4 (CH_2_), 29.8 (CH_2_), 28.8 (CH_2_), 27.2 (CH_2_), 26.1 (CH_2_), 23.6 (CH_2_), 22.9 (CH_3_), 18.2 (CH_3_), and 13.2 (CH_3_) ppm.

### 4.4. Silver Compounds Solution Preparation

Stock solutions of silver lactate (L) at 11.5 mg/mL (in sterile water) and silver deoxycholate:β-Cyclodextrin (B), at 12.29 mg/mL (in DMSO, Sigma-Aldrich, Milan, Italy) were prepared and serially diluted in sterile water to perform in vitro tests. For B dilutions, a maximum of 0.1% of DMSO was present in our conditions and known to have no cytotoxicity [81].

### 4.5. PRP Cluster Preparation

Venous blood from female and male healthy volunteers aged between 20 and 60 years was harvested and utilized for the platelet-rich plasma (PRP) cluster preparation. Other inclusion criteria were non-smokers and the absence of hematologic, neoplastic, and/or infectious diseases, and all subjects gave their informed consent for inclusion before they participated in this study. All the procedures involving human participants were performed in accordance with the ethical standards of the institutional research committee and with the 1964 Helsinki Declaration and its later amendments or comparable ethical standards.

Blood samples were collected in sodium citrate 0.109 M tubes (BD Vacutainer Blood Collection tube^®^, 2.8 mL, BD Italia, Milan, Italy) and centrifuged for 8 min at 560 rcf, 21 °C, without acceleration, and were separated into different blood components: plasma portion in the upper half and a thick layer of plasma with leukocytes and red blood cells in the lower half of the tube. The plasma fraction was collected, in a sterile condition, avoiding the buffy coat, and was frozen at −20 °C before use for at least 24 h. To prepare samples, the frozen plasma aliquot was thawed and centrifuged for 15 min at 2600 rcf, 21 °C, without acceleration, and were broken into pieces of concentrate platelets at the tube’s bottom. The platelet clot was then resuspended in one-third of the plasma total volume in order to triple the physiological platelet concentration. PRP clusters were created in non-tissue culture plates adding to a drop of 50 µL PRP 10 µL of sterile water (PRP) or 10 µL silver/PS/AMB solutions (PRP-Ag)/(PRP-PS/PRP-AMB). Afterwards, 2.5 µL of a sterile calcium chloride solution 456 mM (Merck, Darmstadt, Germany) was also added into the drop, and the content was mixed gently with a pipette before being incubated at 37 °C in 5% CO_2_ for 3 h, during which time the fibrin clot developed.

### 4.6. Scanning Electron Microscopy Observation of PRP and Ag-Enriched PRP Clusters

A dedicated set of non-enriched (CTRL) and Ag-enriched PRP clusters (L: PRP with silver lactate and B: PRP with silver deoxycholate:β-Cyclodextrin) were fixed in Karnowsky’s solution (4% paraformaldehyde + 2.5% glutaraldehyde in 0.1 M cacodylate buffer pH 7.4) for 30 min at 4 °C. After washing in a cacodylate buffer, cells were dehydrated in ethanol (50–100%) and then hexamethyldisilazane [82].

Dried samples were then coated with a 10 nm layer of gold using a sputter coater (Emitech K500X, Quorum Technologies, Laughton, UK) to improve their electrical conductivity and thermal stability. The observations were performed using a Quanta 200 (FEI-Philips, Eindhoven, The Netherlands) scanning electron microscope (SEM) in high-vacuum mode using a 10 keV electron beam energy. A set of representative images was collected for each sample in the range from 100× to 10,000×. The microstructural architecture of the clusters was investigated in terms of platelet distributions and fibrin fiber arrangement according to previous experience [56] and the literature [55,83].

### 4.7. Antimicrobial Activity on Planktonic Cells

All the strains used in this study were obtained from the American Type Culture Collection (ATCC, Manassas, VA, USA). Gram-positive *Staphylococcus aureus* ATCC 25923, *Staphylococcus epidermidis* ATCC 35984, and yeast *Candida albicans* ATCC 10231 were seeded from frozen stocks onto Tryptic Soy Agar (TSA, Scharlab, Barcelona, Spain) or Sabouraud Dextrose Agar (SDA, Scharlab, Barcelona, Spain), respectively, and incubated overnight at 37 °C.

The antimicrobial activity of Ag solutions or PRP-Ag clusters against Gram-positive *S. aureus* ATCC 25923 and *S. epidermidis* ATCC 35984 planktonic cells or yeast *C. albicans* ATCC 10231 planktonic cells was evaluated by the 3-[4,5-dimethylthiazol-2-yl]-2,5-diphenyltetrazolium bromide (MTT)-based assay.

Briefly, bacterial suspensions at the concentration of 5 × 10^5^ colony-forming units (CFUs)/mL were prepared in Mueller–Hinton Broth (Scharlab, Barcelona, Spain). Fungal suspensions at the concentration of 0.5–2.5 × 10^5^ CFUs/mL were prepared in Roswell Park Memorial Institute (RPMI) 1640 (Sigma–Aldrich, Milan, Italy) buffered with MOPS (Sigma–Aldrich, Milan, Italy) and supplemented with 2% glucose (Biolife, Monza, Italy); pH 7.0 (RPMI+2%G). Ag solutions (10 µL) or PRP-Ag were co-incubated with 200 µL of microbial suspensions at 37 °C.

Negative controls consisted of a PS solution (10 µL)/PRP-PS for staphylococcal strains or an AMB solution (10 μL)/PRP-AMB for *C. albicans*, whereas positive controls consisted of water (10 µL)/PRP only.

After 24 h, microbial cells were harvested by centrifugation at 12,000 rpm for 15 min at 4 °C, resuspended in 200 µL of an MTT working solution [(0.075% MTT (Scharlab, Barcelona, Spain)] supplemented with 0.1% glucose (Biolife, Monza, Italy) and 10 μM menadione (Sigma–Aldrich, Milan, Italy), and incubated at 37 °C for 10 min (*S. aureus*, *S. epidermidis*) or 40 min (*C. albicans*).

Subsequently, microbial cells were collected by centrifugation at 12,000 rpm for 15 min at 4 °C, and formazan crystals were dissolved with 200 µL of a lysis buffer solution (LB) [dimethyl sulfoxide (Scharlab, Barcelona, Spain)/0.1 M glycine pH 10.2 (Sigma–Aldrich, Milan, Italy) (7:1)]. The absorbance of the obtained solutions was measured at 570 nm (Victor^3^V^TM^, Perkin Elmer, Milan, Italy). Data were normalized to the background and expressed as percentages of microbial growth, considering the mean value of the positive control as 100% vitality.

### 4.8. Activity against Biofilm Formation

The inhibitory activity of PRP-Ag clusters against Gram-positive *S. aureus* ATCC 25923, *S. epidermidis* ATCC 35984, or yeast *C. albicans* ATCC 10231 biofilm formation was evaluated by the MTT-based assay. Biofilm formation was carried out as described in Ceresa et al. [84,85].

Briefly, bacterial suspension at the concentration of 1 × 10^7^ CFUs/mL was prepared in Tryptic Soy Broth (Scharlab, Barcelona, Spain) supplemented with 1% glucose (Scharlab, Barcelona, Spain). Fungal suspensions at the concentration of 1 × 10^6^ CFUs/mL were prepared in RPMI+2%G; pH 7.0. PRP-Ag were co-incubated with 200 µL of microbial suspensions at 37 °C for 24 h. Negative controls, PRP-PS for bacteria or PRP-AMB for fungal strains, and the positive control (PRP only), were also included.

Biofilms were washed two times with 200 µL of PBS, dipped with 200 µL of an MTT working solution, and incubated at 37 °C for 10 min (*Staphylococcus* spp.) or 40 min (*C. albicans*).

Afterwards, formazan crystals formed by metabolic active cells within biofilms were dissolved with 200 µL of LB, and the absorbance of the obtained solutions was measured at 570 nm. Data were normalized to the background and expressed as percentages of formed biofilm, considering the mean value of the positive control as 100% vitality.

### 4.9. Isolation of hOBs from Human Trabecular Bone Fragments

Human primary osteoblasts were isolated from the trabecular bone of adult femoral heads, provided by the Orthopaedic and Traumatology Unit at the hospital “Maggiore della Carità”, Novara, Italy. The samples represent surgical discharge materials and, therefore, their use does not need ethics committee approval. Written informed consent, specifying that residual material destined to be disposed of could be used for research, was signed by each participant before the biological materials were removed, in agreement with Rec(2006)4 of the Committee of Ministers Council of Europe on research on biological materials of human origin.

After being minced and washed in PBS, bone fragments were digested with collagenase/elastase as described in Bosetti et al. [86] and plated with Iscove’s modified Dulbecco’s medium (IMDM, Euroclone, Milan, Italy) supplemented with 10% fetal bovine serum (FBS, Hyclone GE Healthcare, Logan, UT, USA), 2 mM L-glutamine (L-Glu, Sigma-Aldrich, Milan, Italy), and PS at 37 °C in 5% CO_2_. Cells appeared within 10 days and were used for all the experiments up to the seventh passage after being characterized by alkaline phosphatase expression [87].

### 4.10. PRP-Ag Cluster Biocompatibility on hOBs

The biocompatibility of PRP-Ag clusters on hOBs was evaluated using the MTT-based assay. Osteoblasts were plated at a cell density of 10^4^ cells/well in 48-well plates in 500 µL of IMDM supplemented with 10% FBS, 2 mM L-Glu, and PS. After 24 h, PRP-Ag clusters were added to wells, and plates were incubated for a further 24 h at 37 °C in 5% CO_2_. PRP clusters were used as a positive control of cell viability, whereas negative controls consisted of cells treated with Triton-X 1% *v*/*v* (Sigma-Aldrich, Milan, Italy).

After 24 h, clusters were removed, and 50 µL of the MTT (Sigma-Aldrich, Milan, Italy) solution (5 mg/mL in PBS) was added to each well. After 3 h at 37 °C in 5% CO_2_, media were removed, and formazan salts formed were eluted with 200 µL/well of a 1 M HCl/isopropanol (1:20) solution for 20 min at 37 °C. The absorbance of the obtained solutions was measured at 570 nm, and data were normalized to the background and expressed as percentages of hOB viability considering the mean value of PRP as 100% vitality.

### 4.11. PRP-Ag Differentiation Activity on hOBs

The differentiation activity of PRP-Ag clusters on hOBs was investigated in 48-well plates. Cells (1.0 × 10^4^ cells/well) were seeded and incubated for 24 h; afterwards, PRP-Ag clusters were gently added to each well to avoid cell detachment. PRP clusters were included as internal controls, whereas not-treated hOBs represented the basal control. Furthermore, PRP-DEX (10 nM, Sigma-Aldrich, Milan, Italy) clusters were used as a positive control of differentiation. Experiments were carried out until 6 and 12 days of culture. The differentiation fate of hOBs was evaluated by ALP staining and ATP quantification as follows. After cluster and media removal, cells were washed with PBS and incubated with a lysis buffer solution (0.05 M Tris-HCl, SDS 0.05% *v*/*v* pH 8) at 50 °C for 10 min. One hundred microliters of lysate were mixed with 100 µL of the ALP substrate solution (para-nitrophenyl–phosphate 5 mg/mL in 0.25 M Tris-HCl pH 9.5 and 10 mM MgCl_2_) and incubated for 1 h at 37 °C. Afterwards, the absorbance of p-nitrophenol was read at 405 nm in a microplate spectrophotometer (VICTOR^3^V^TM^, PerkinElmer Inc., Waltham, MA, USA), and data were normalized to the background. To quantify the number of cells, the ATP (ViaLight^TM^ Plus kit, Lonza, Rockland, ME, USA) test was performed at the same time points following the kit instructions. Normalized ALP data with ATP were calculated as ALP/ATP*10,000.

### 4.12. Statistical Analysis

All the experiments were carried out in triplicate and repeated four times (n = 12). Quantitative data were shown as mean values and standard deviations. Graphs and statistical analysis were performed using Prism 8 software (GraphPad Software, San Diego, CA, USA). Data were analyzed using a one-way ANOVA and a two-way ANOVA followed by Tukey’s post hoc test. Results were considered to be statistically significant when *p* < 0.05.

## 5. Conclusions

In this study, we have suggested a new strategy based on platelet-rich plasma clusters enriched with silver to exploit autologous blood derivate, preventing implant-associated infections and enhancing bone healing. Thanks to its excellent safety profile, silver lactate can be used at the highest concentration, exerting an excellent inhibitory effect on fungal and Gram-positive strains. Silver deoxycholate:β-Cyclodextrin, demonstrates superior antimicrobial efficacy and stimulatory activity on osteoblast differentiation, allowing its use at lower concentrations.

Taking into account the results obtained on antimicrobial effectiveness and eukaryotic cell biocompatibility, a satisfactory balance has been obtained for PRP-L at 0.3 µg/mm^2^ and PRP-B at 0.2 µg/mm^2^. Both clusters possess a high antibacterial and antifungal activity against *S. aureus*, *S. epidermidis*, and *C. albicans* planktonic cells and a marked antibiofilm effect without negatively affecting bone cell activity and survival. The enhanced efficacy of B can likely be attributed to both the higher potency of the compound itself and a greater quantity of silver bound to fibrin clot, suggesting that the rationale behind the synthesis of silver deoxycholate:β-Cyclodextrin has been appropriate and has proven to be effective to achieve this study goal.

Hence, our study presents not only a novel strategy for utilizing autologous blood derivatives as antimicrobial agents but also introduces a new compound that warrants further exploration. A parallel in vivo assessment is crucial for a more comprehensive understanding of the implications of silver application in a complex environment, like bone tissue. The local application of antimicrobial therapy is not meant to replace systemic antibiotic administration but rather enables a reduction in the dosage of these antibiotics thanks to the synergistic exploitation of both local and systemic antimicrobial actions.

Overall, these results expand the body of evidence that boosts additional testing toward other applications of PRP-Ag in fields where PRP is already commonly used. Among possible relevant scenarios, PRP-Ag could be potentially employed in wound healing or general surgery, where a 3D scaffold rich in growth factors is needed and microorganism colonization occurs early, frequently, and extensively.

## Data Availability

The datasets used and analyzed during the current study are available from the corresponding author upon reasonable request.

## Supplementary Materials

The following supporting information can be downloaded at: https://www.mdpi.com/article/10.3390/ijms25094842/s1.
