# Supplementary material for: An In Vitro Study on the Application of Silver-Doped Platelet-Rich Plasma in the Prevention of Post-Implant-Associated Infections
Source: Int J Mol Sci. 2024 Apr 29;25(9):4842. doi: 10.3390/ijms25094842 (PMC11084394; doi:10.3390/ijms25094842)
Supplement: Supplementary file 1 [file ijms-25-04842-s001.zip › ijms-2953906-supplementary.pdf]

Supplementary Figures

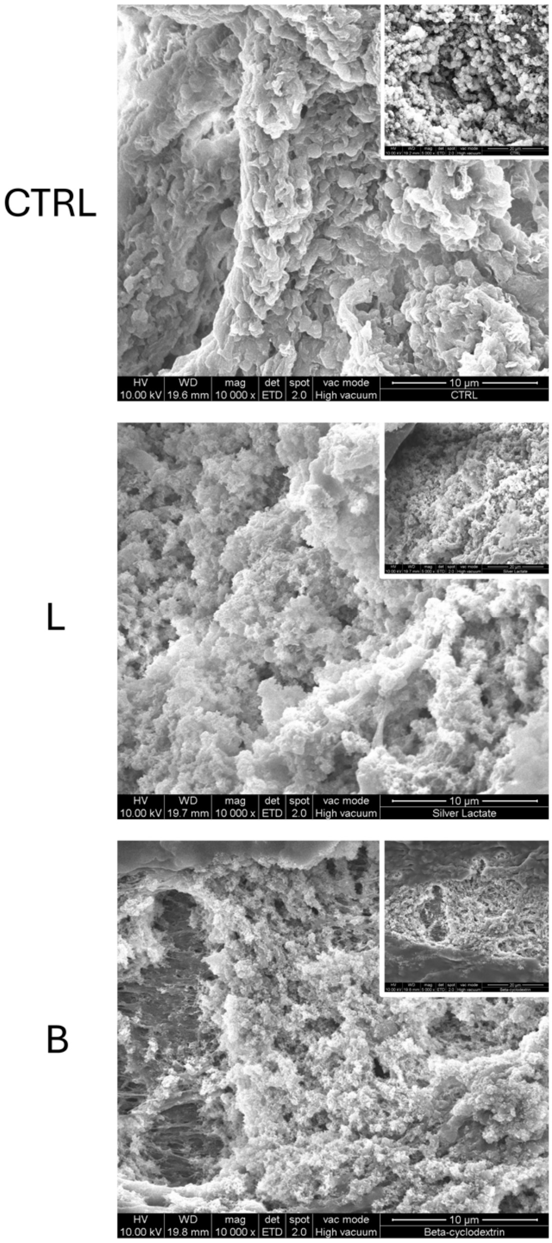

Supplementary Figure S1. Microstructure of PRP and Ag-enriched PRP clusters. CTRL: non-enriched PRP; **L**: Ag-enriched PRP with silver lactate; **B**: Ag-enriched PRP with silver deoxycholate:β-Cyclodextrin. Representative images were obtained by scanning electron microscopy in high-vacuum mode. Original magnification: 10000× (insets 5000×).

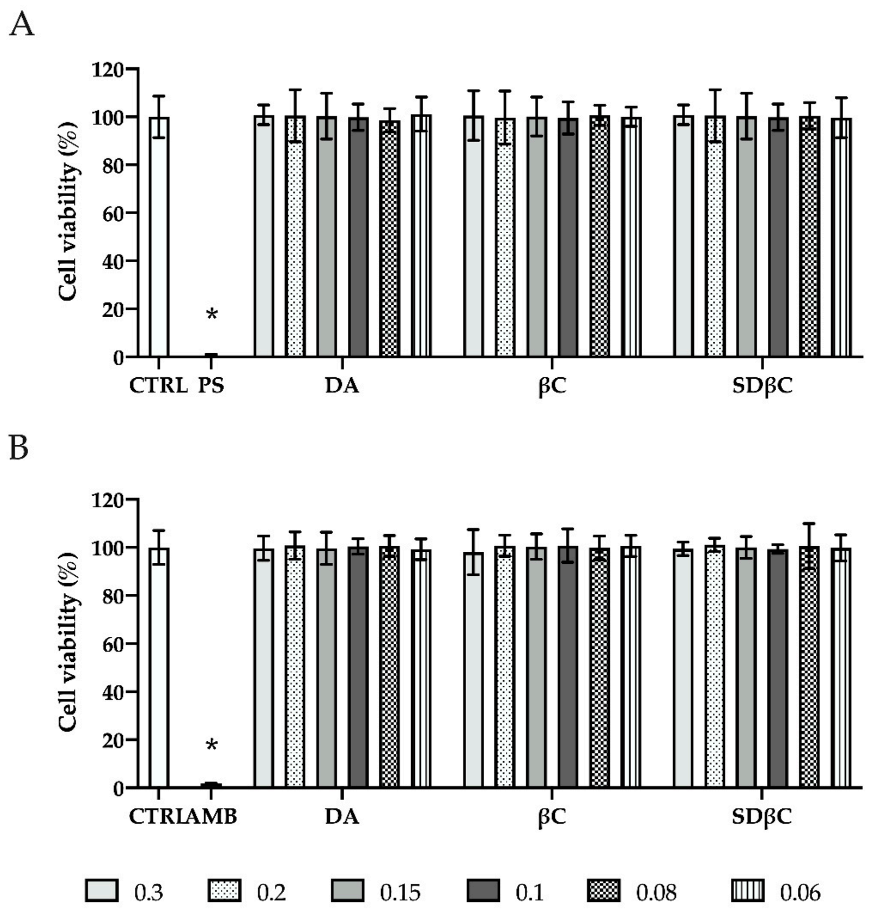

Supplementary Figure S2. Effect of deoxycholic acid, β-cyclodextrin, sodium deoxycholate: β-cyclodextrin on viability of microbial planktonic cells. The metabolic activity of *S. aureus* (A) and *C. albicans* (B) planktonic cells was evaluated after 24h of incubation through MTT assay. Data are expressed as percentage of cell viability, considering CTRL (positive control) as 100% vitality. Pen Strep (PS) or Amphotericin B (AMB) were used as negative controls. CTRL: not-treated microbial cells; PS: treatment with PS; AMB: treatment with AMB; DA: treatment with deoxycholic acid; **βC**: treatment with β-cyclodextrin; SD**βC**: treatment with sodium deoxycholate: β-cyclodextrin**.** Error bars represent standard deviation, *$p<0.0001$ significantly different from CTRL.

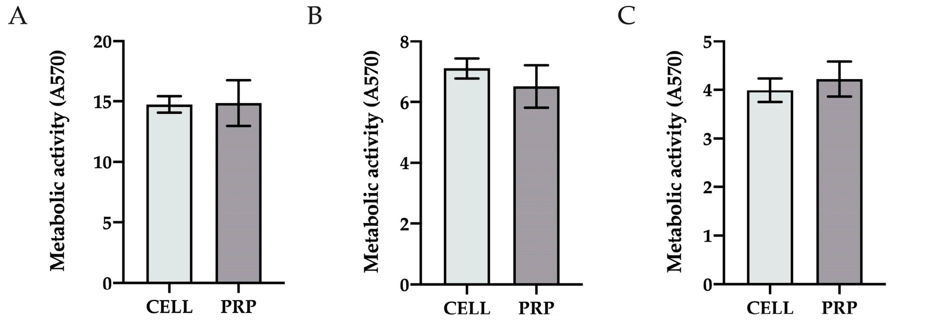

Supplementary Figure S3. Effect of PRP on viability of microbial planktonic cells. The metabolic activity of *S. aureus* (A), *S. epidermidis* (B) and *C. albicans* (C) planktonic cells was evaluated after 24h of incubation through MTT assay. Data are expressed as mean value of cell viability and error bars represent standard deviation. CELL: not-treated microbial cells; PRP: treatment with PRP.
